# Bizarre parosteal osteochondromatous proliferation: an educational review

**DOI:** 10.1186/s13244-023-01455-0

**Published:** 2023-06-19

**Authors:** Salvatore Gitto, Francesca Serpi, Carmelo Messina, Domenico Albano, Andrea Di Bernardo, Elisabetta Armiraglio, Luca Cannavò, Simone Mazzoli, Alessandro Luzzati, Antonina Parafioriti, Luca Maria Sconfienza

**Affiliations:** 1 IRCCS Istituto Ortopedico Galeazzi, via Cristina Belgioioso 173, 20157 Milan, Italy; 2 Dipartimento di Scienze Biomediche per la Salute, Università degli Studi di Milano, Milan, Italy; 3 Pathology Department, ASST Pini-CTO, Milan, Italy

**Keywords:** Bizarre parosteal osteochondromatous proliferation, Cartilaginous tumor, Periostitis, Nora lesion, Osteochondroma

## Abstract

Bizarre parosteal osteochondromatous proliferation (BPOP) is a surface-based bone lesion belonging to the group of benign chondrogenic tumors. The aim of this review is to familiarize the readers with imaging features and differential diagnosis of BPOP, also addressing pathological presentation and treatment options. The peak of incidence of BPOP is in the third and fourth decades of life, although it can occur at any age. Hands are the most common location of BPOP (55%), followed by feet (15%) and long bones (25%). On imaging, BPOP appears as a well-marginated mass of heterotopic mineralization arising from the periosteal aspect of the bone. Typical features of BPOP are contiguity with the underlying bone and lack of cortico-medullary continuity, although cortical interruption and medullary involvement have been rarely reported. Histologically, BPOP is a benign bone surface lesion characterized by osteocartilaginous proliferation with disorganized admixture of cartilage with bizarre features, bone and spindle cells. Differential diagnosis includes both benign—such as florid reactive periostitis, osteochondroma, subungual exostosis, periosteal chondroma and myositis ossificans—and malignant lesions—such as periosteal chondrosarcoma and surface-based osteosarcoma. Treatment consists of surgical resection. Local recurrences are common and treated with re-excision.

**Critical relevance statement** Bizarre parosteal osteochondromatous proliferation is a benign mineralized mass arising from the periosteal aspect of bone cortex. Multi-modality imaging characteristics, pathology features and differential diagnosis are here highlighted to familiarize the readers with this entity and offer optimal patient care.

## Key points

- Bizarre parosteal osteochondromatous proliferation (BPOP) is a benign surface-based bone lesion.
- Hands are the most common location, followed by long bones and feet.
- BPOP is a mineralized mass arising from the periosteal aspect of bones.
- Histologically, it is composed of a mixture of cartilage, bone, fibrous tissue.
- Treatment consists of surgical resection, but local recurrences are common.

## Background

Bizarre parosteal osteochondromatous proliferation (BPOP) was first described by Nora in 1983 and was previously known as “Nora’s lesion” [1]. The term “Nora’s lesion” is not recommended according to the 2020 World Health Organization classification of bone tumors, where BPOP is grouped within the benign chondrogenic tumors [2]. BPOP is described as a rare benign surface-based bone lesion [1]. However, its true incidence is difficult to assess, as most lesions are described in case reports or series and a few larger studies which suffer from retrospective design. Hence, BPOP may be a potentially unknown entity among many radiologists, particularly in institutions without a dedicated focus on bone tumors. The aim of this review is to familiarize the readers with imaging features and differential diagnosis of BPOP, also addressing pathological presentation and treatment options, thus increasing awareness of this entity.

## Epidemiology and clinical presentation

The peak of incidence of BPOP is in the third and fourth decades of life, although it can occur at any age [3]. There is no gender predilection [3]. Etiology is currently unknown. A possible traumatic etiology has been suggested [4]. Particularly, BPOP has been viewed by some authors as the intermediate stage of a spectrum of reactive lesions, which also encompasses florid reactive periostitis (early stage) and acquired osteochondroma or turret exostosis (late stage) [4]. More recently, other authors have proposed that BPOP may represent a neoplastic rather than reactive lesion [5]. This notion is supported by the identification of recurrent cytogenetic abnormalities associated with BPOP [6].

BPOP presents clinically as a slow-growing firm mass, which is usually painless and may cause local symptoms due to mass effect [3]. Most lesions measure 1–3 cm [2]. Hands are the most common location of BPOP, accounting for 55% of cases in the largest case series to date [3], with a tendency to occur in the phalanges [7]. Particularly, in the hands, BPOP was found in the phalanges and metacarpals in 92% and 8% of cases, respectively, and favored metaphyseal and diaphyseal locations [7]. BPOP also occurs in feet and long bones in 15% and 25% of cases, respectively, most commonly at the metaphysis [3]. Other locations, such as the skull [3] and facial bones [8], are rare.

## Imaging features

According to the largest radiology-based study to date, BPOP can be defined as a “well-marginated mass of heterotopic mineralization arising from the periosteal aspect of an intact cortex, without medullary changes” [7]. Some atypical imaging features representing exceptions to this general definition have been described and are detailed below. Imaging appearance also varies according to maturation of BPOP [7].

**Fig. 1:**
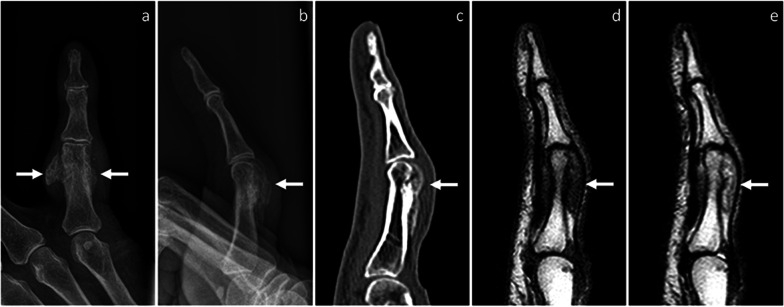
BPOP arising from the proximal phalanx of the little finger. On X-rays, frontal (**a**) and lateral, (**b**) views show a well-defined mass of heterotopic mineralization, which is contiguous to the proximal phalanx. On sagittal CT image (**c**), the mass is cortex-based with no cortico-medullary continuity, cortical breakthrough, or marrow extension. On MRI, the mass is hypointense on T1-weighted (**d**) and hyperintense on T2-weighted (**e**) sagittal sequences, respectively. Location and imaging findings are in keeping with BPOP. Surgical resection was performed and BPOP was pathologically proven. Arrows point at BPOP in all images

**Fig. 2:**
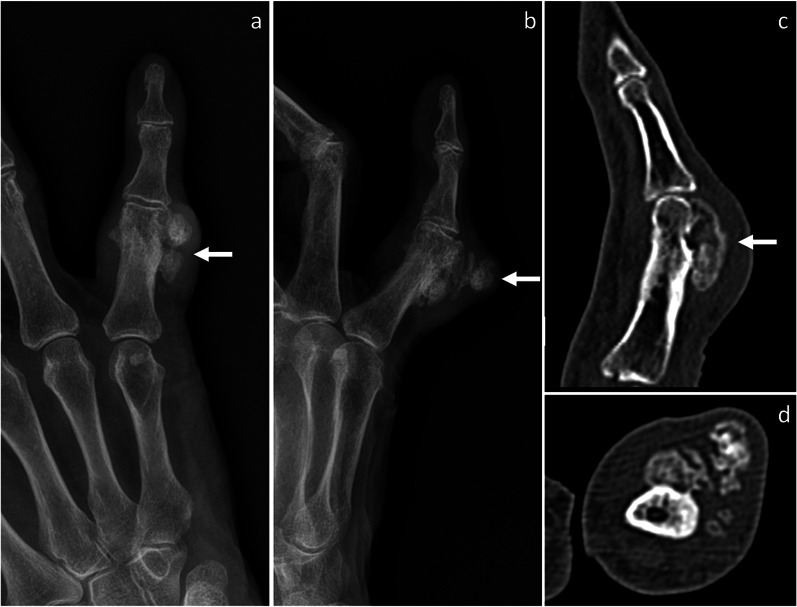
Recurred BPOP arising from the proximal phalanx of the little finger (same patient shown in Fig. 1). Eighteen months after surgery, BPOP recurrence is noted and shows more irregular mineralization compared to the original lesion, as shown on frontal (**a**) and oblique (**b**) X-rays views, as well on sagittal (**c**) and axial (**d**) CT images. Arrow points at BPOP in all images

**Fig. 3:**
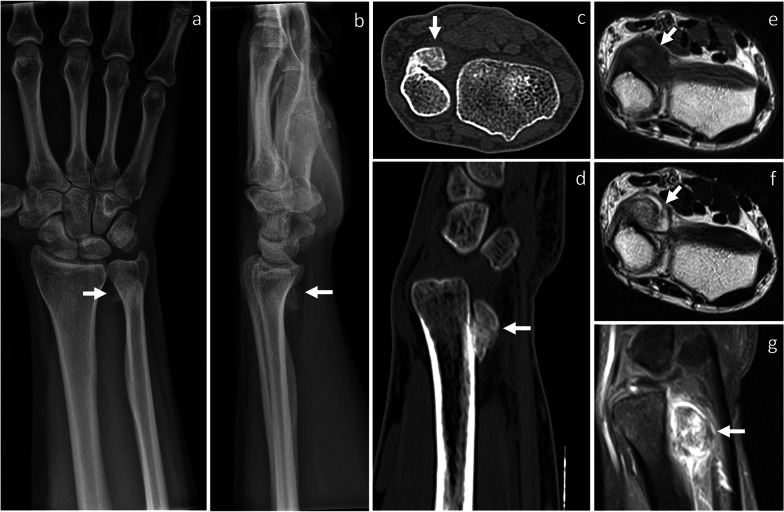
BPOP arising from the distal ulnar metaphysis. On X-rays, frontal (**a**) and lateral (**b**) views show a well-defined mass of heterotopic mineralization, which is contiguous to the palmar aspect of the distal ulnar metaphysis. On axial (**c**) and sagittal (**d**) CT images, the mass is cortex-based with no cortico-medullary continuity, cortical breakthrough, or marrow extension. On axial T1-weighted (**e**) and T2-weighted (**f**) MRI sequences, the mass shows low-to-intermediate and high signal, respectively. After contrast administration, marked contrast enhancement is seen on sagittal fat-saturated T1-weighted sequence (**g**). After biopsy, surgical resection was performed and BPOP was pathologically proven. Arrow points at BPOP in all images

**Fig. 4:**
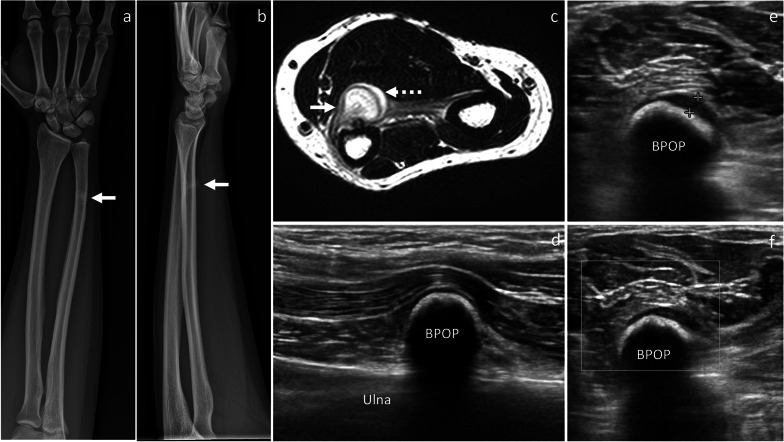
BPOP arising from the distal ulnar diaphysis. On X-rays, frontal (**a**) and lateral (**b**) views show a mineralized mass, which is contiguous to the distal diaphysis of the ulna. On axial T2-weighted MRI sequence (**c**), the mass is hyperintense with higher signal at the periphery representing cartilage covering (dashed arrow). Longitudinal B-mode ultrasound image (**d**) depicts a calcified mass, which is contiguous to the palmar aspect of the ulna and impinges on the flexor muscles. Axial B-mode ultrasound image (**e**) shows a thin hypoechoic layer (calipers) superficial to the mineralized mass, which is in keeping with cartilage covering. No increased vascularity is seen on power Doppler imaging (**f**). After biopsy, surgical resection was performed and BPOP was pathologically proven. White arrow points at BPOP in all images

On radiographs, a periosteal soft-tissue swelling can be noted in the early stages, possibly with tiny calcifications. This mass shows progressive mineralization and becomes partially or completely ossified over several months. In the late stages, BPOP presents as sessile or pedunculated heterotopic bone formation, which is contiguous to the underlying bone cortex (Figs. 1, 2, 3, and 4) [7].

**Fig. 5:**
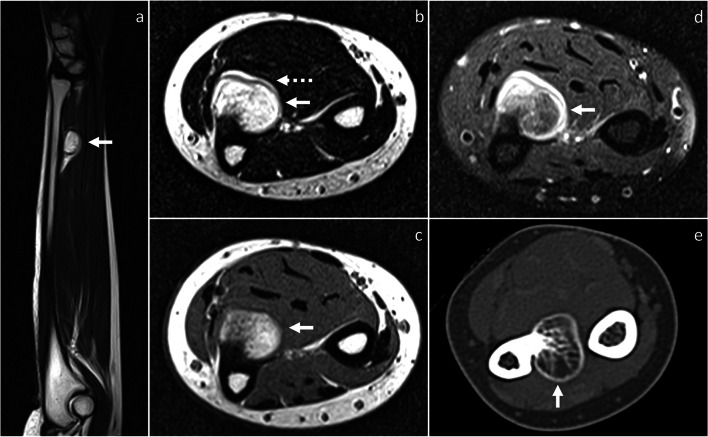
Recurred BPOP arising from the distal ulnar diaphysis (same patient shown in Fig. 4). Three years after surgery, BPOP recurrence presents as a cortex-based completely ossified mass, which is contiguous to the palmar-radial aspect of the ulna. On MRI, the ossified component of the mass is hyperintense on both T2-weighted (**a**, **b**) and T1-weighted (**c**) sequences. After contrast administration, contrast enhancement is noted on fat-saturated T1-weighted sequence (**d**). No cortical breakthrough is seen on CT (**e**). White arrow points at BPOP in all images. Dashed arrow points at the outermost cartilaginous layer of BPOP in (**b**)

CT shows the same features of BPOP as described on radiographs, with better anatomical detail. Particularly, in the late stages, an ossified surface-based mass is depicted in contiguity but no continuity with the underlying cortex (Figs. 1, 2, 3 and 5). No evidence of cortical breakthrough or bone marrow extension is typically seen [7, 9]. However, atypical findings of cortical destruction [10] and cortico-medullary continuity with the underlying bone [11, 12] have been reported.

On MRI, BPOP shows low-to-intermediate signal on T1-weighted sequences and intermediate-to-high signal with hyperintensity at the periphery representing cartilage on T2-weighted sequences, respectively. After intravenous contrast administration, contrast enhancement is seen [13]. Normal cortical appearance and absent marrow signal changes are typical features of BPOP and well depicted on MRI (Figs. 1, 3, 4, and 5) [13]. However, atypical findings of cortico-medullary continuity with the underlying bone [11, 12], cortical invasion [14, 15] and reactive signal changes in the adjacent bone marrow and soft-tissues [16] have been reported.

On ultrasound, BPOP presents as a calcified soft-tissue mass, which has no continuity with the underlying bone cortex (Fig. 4) [7].

## Differential diagnosis

Accurate imaging evaluation is crucial for guiding patient care, as imaging features of BPOP may mimic other benign and aggressive surface-based bone lesions. Patient age, lesion location and main imaging characteristics useful for differentiating these entities from BPOP are discussed below.

**Fig. 6:**
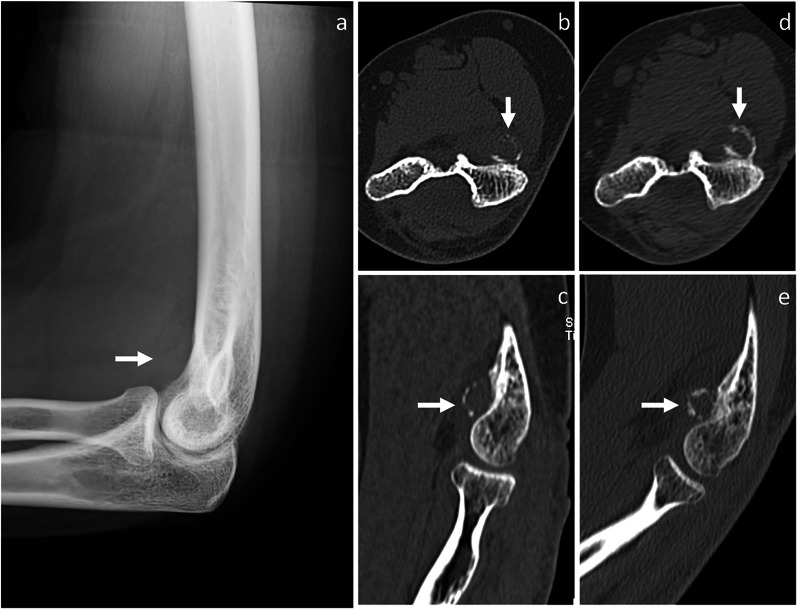
Florid reactive periostitis. On lateral X-rays view (**a**), a soft-tissue swelling is noted in contiguity with the volar aspect of the distal humerus. On axial (**b**) and sagittal (**c**) CT images, a partially mineralized mass with peripheral calcifications and mild periosteal thickening is noted. No cortical discontinuity is seen. Three months later, peripheral calcifications become more prominent, as shown in axial (**d**) and sagittal (**e**) CT images. Florid reactive periostitis ossificans was pathologically proven. Arrow points at the lesion in all images

*Florid reactive periostitis (also known as fibro-osseous pseudotumor of digits)* It has been viewed by some authors as the early stage of a spectrum of reactive lesions, which could mature into BPOP [4]. However, according to the 2020 World Health Organization classification of bone tumors, BPOP is considered as a separate entity and possible neoplastic etiology is suggested [2]. Like BPOP, florid reactive periostitis occurs most commonly in the phalanges of the hands and feet and, less frequently, in metacarpals, metatarsals and long bones [5]. It is mostly seen in young adults ranging in age from 20 to 40 years [5], similar as BPOP. On imaging, a soft-tissue swelling with heterotopic ossification is seen in contiguity with an intact bone cortex [17, 18], although cortical destruction has been occasionally reported [18]. Periosteal reaction is usually present [17]. Over weeks to months, periosteal reaction maturation can occur and result in a soft-tissue calcified shadow, which is also known as florid reactive periostitis ossificans (Fig. 6) [5].

**Fig. 7:**
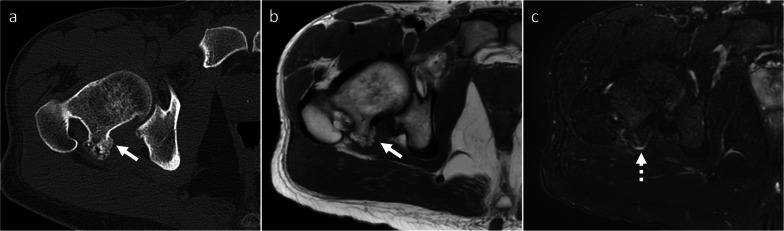
Osteochondroma. On axial CT (**a**) and T1-weighted MRI (**b**) images, osteochondroma (white arrow) exhibits cortico-medullary continuity with the underlying native bone. On axial fat-saturated T2-weighted MRI sequence, the lesion shows a thin and uniform cartilaginous cap (dashed arrow). Osteochondroma was pathologically proven

*Osteochondroma* It is a cartilage-capped osseous exophytic lesion arising from metaphysis or metaphyseal equivalents, which can be either sessile (broad base exceeding its length) or pedunculated (narrow base exceeded by its length) [19]. It is usually discovered in the first three decades of life. Osteochondroma is mostly located in long bones, particularly femur and tibia. Solitary osteochondroma rarely occurs in the hands and feet [19]. Unlike BPOP (with typical features), osteochondroma exhibits cortico-medullary continuity with the underlying native bone (Fig. 7). Additionally, it has an uniform cartilaginous cap, which may show calcifications and differs from the disorganized cartilage covering observed in the outermost layer of BPOP [19]. Cortico-medullary continuity and cartilaginous cap are best evaluated on CT and MRI, which may also depict enlargement of the cartilaginous cap (> 1.5 cm after skeletal maturity) suggesting degeneration to secondary peripheral chondrosarcoma [20]. Finally, osteochondroma is usually oriented away from the nearest joint and extends parallel to the long axis of the native bone [19].

**Fig. 8:**
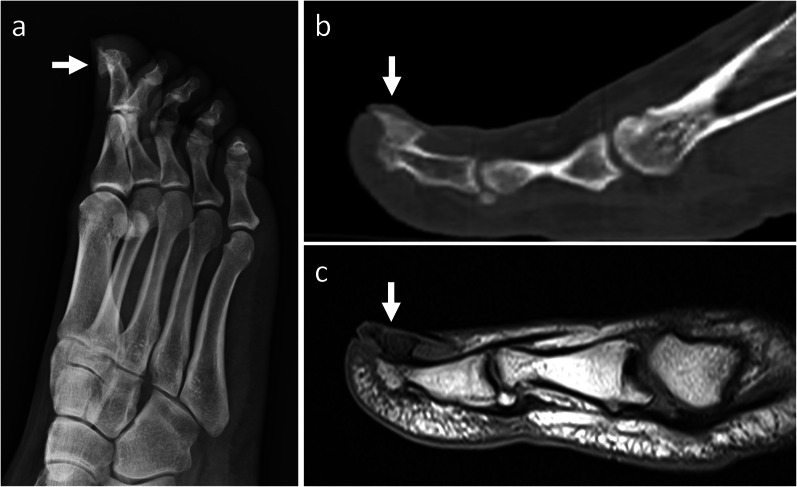
Subungual exostosis. On X-rays (**a**), a mineralized mass is noted in contiguity with the dorsal aspect of the distal phalanx of the big toe. On sagittal CT (**b**) and T1-weighted MRI (**c**) images, no cortical or marrow continuity with the underlying bone is seen. Subungual exostosis was pathologically proven. Arrow points at the lesion in all images

*Subungual exostosis* It is an osteocartilaginous proliferation arising from the distal phalanx of the fingers or toes [5]. It commonly presents in adolescents and young adults [5], similar as BPOP. On imaging, subungual exostosis appears as a bony outgrowth that projects from the dorsal aspect of the terminal phalanx. Unlike osteochondroma, subungual exostosis reveals no cortical or marrow continuity with the underlying bone (Fig. 8) [21].

**Fig. 9:**
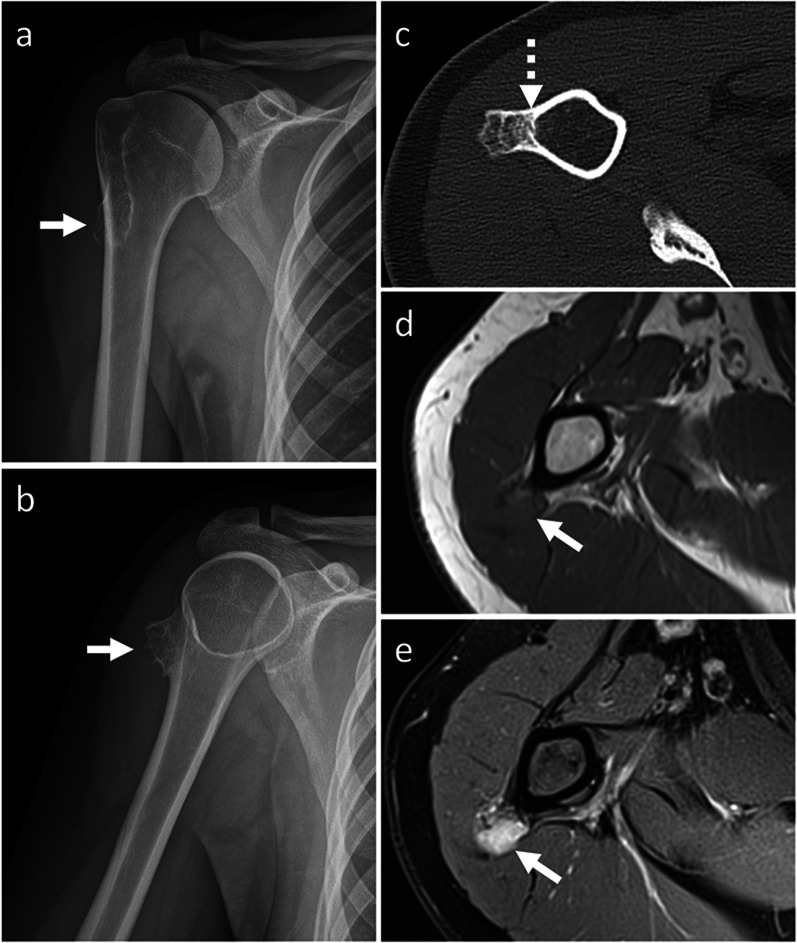
Periosteal chondrosarcoma. On X-rays (**a**, **b**), a mineralized surface-based mass of the proximal humerus is seen. On axial CT image (**c**), the mass is cortex-based and partially ossified. Cortical remodeling and erosion (dashed arrow) are noted. On axial T1-weighted (**d**) and proton density-weighted (**e**) MRI sequences, the mass shows low and high signal, respectively. No marrow or soft-tissue extension is noted. Periosteal chondrosarcoma was pathologically proven. White arrow points at the lesion in all images

*Periosteal chondroma and periosteal chondrosarcoma* Periosteal chondroma and chondrosarcoma are cartilage-forming tumors arising from metaphysis and diaphysis of tubular bones [22]. They are mostly located in the humerus and femur [22]. Less frequently, periosteal chondroma is found in the hands [23]. Periosteal chondroma and chondrosarcoma often occur in young adults, similar as BPOP [22, 23]. Size is the most useful imaging feature to differentiate between periosteal chondroma and chondrosarcoma, which are usually smaller and larger than 3 cm, respectively [19, 24]. They are both lobular iuxta-cortical masses showing “rings and arcs” calcifications on radiographs and CT, as well as high signal on T2-weighted sequence, low signal on T1-weigthed sequence and peripheral and septal contrast enhancement on MRI, which are in keeping with chondroid matrix [19, 24]. However, these findings are subtle compared to centrally located lesions. Unlike BPOP, periosteal chondroma and periosteal chondrosarcoma exhibit saucerization and sclerotic margination of the underlying cortex, as well as dense periosteal reaction. Metaplastic ossification, aggressive periosteal reaction like Codman triangle, cortical invasion, intramedullary extension and tendency to permeate into the adjacent soft tissues are suggestive of periosteal chondrosarcoma rather than chondroma (Fig. 9) [19, 24]. However, imaging features of periosteal chondroma and chondrosarcoma often overlap, and tissue sampling is then required for definitive diagnosis.

**Fig. 10:**
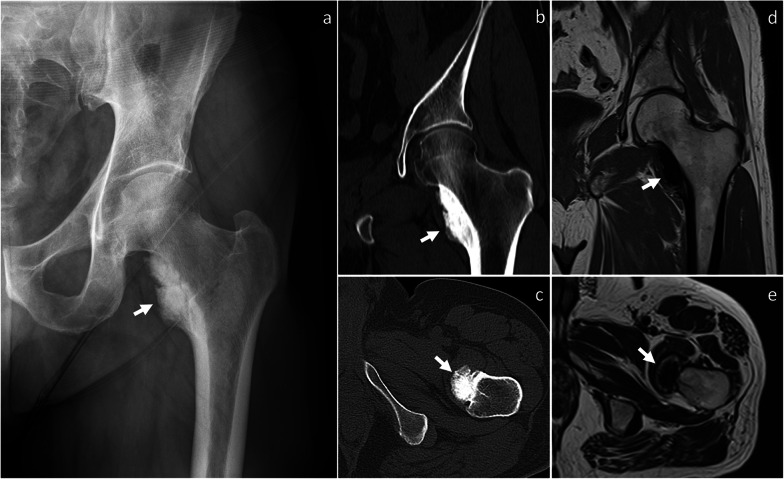
Parosteal osteosarcoma. On X-rays (**a**), an exophytic heavily ossified mass is seen in contiguity with the medial aspect of the femoral neck. On coronal (**b**) and axial (**c**) CT images, the mass is denser centrally than at the periphery. The mass shows predominantly low signal representing mineralized component on both coronal T1-weighted (**d**) and axial T2-weighted (**e**) MRI sequences. Parosteal osteosarcoma was pathologically proven. Arrow points at the lesion in all images

*Surface-based osteosarcoma* Surface osteosarcoma includes parosteal, periosteal and high-grade surface osteosarcoma [2]. Periosteal and high-grade surface osteosarcomas are of intermediate and high-grade, respectively, and show aggressive features such as aggressive periosteal reaction (periosteal variant), large circumferential cortical involvement (≥ 50%), cortical erosion and medullary involvement (high-grade surface variant) [24], which help in differentiating them from BPOP. Conversely, parosteal osteosarcoma is of low-grade [24] and may mimic BPOP. It most commonly affects patients in the second-to-fourth decades, similar as BPOP [24]. It has predilection for long bones and is typically metaphyseal in location or, occasionally, diaphyseal or meta-diaphyseal [24]. Unlike BPOP, parosteal osteosarcoma rarely occurs in the hands [25] and feet [26]. Parosteal osteosarcoma presents as a heavily ossified lobular exophytic mass with soft-tissue component, which is denser centrally than at the periphery (Fig. 10). An incomplete and irregular cartilaginous covering may infrequently be present. Unlike BPOP (with typical features), it can be associated with cortical erosion and medullary involvement. Periosteal reaction is uncommon unless dedifferentiation occurs [24].

**Fig. 11:**
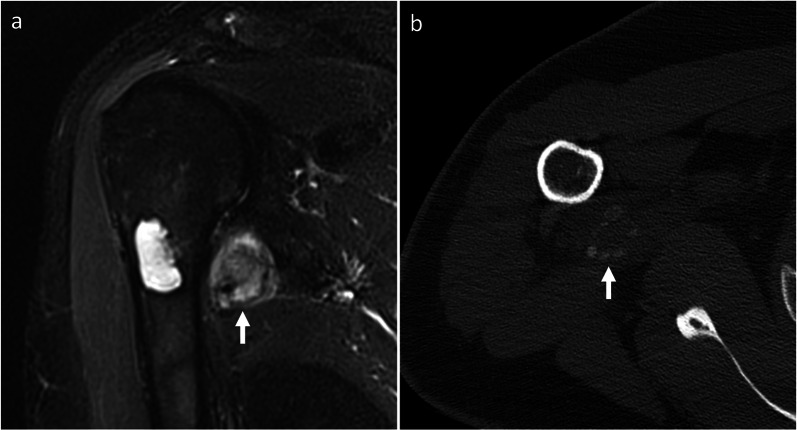
Myositis ossificans. On coronal fat-saturated T2-weighted MRI sequence (**a**), an intermediate-to-high signal mass is noted close to the medial aspect of the proximal humerus. This mass is separated from the underlying bone. A central chondroid lesion of the humerus is also seen. On CT (**b**), “zonal phenomenon” with peripheral mineralization and central lucency is shown. Myositis ossificans was pathologically proven. Arrow points at myositis ossificans in both images

*Myositis ossificans* It is a tumor mimicker resulting from heterotopic bone formation, typically following trauma [27]. It can occur at any age. Unlike BPOP, ossification progresses from periphery to center. Thus, the major distinguishing feature of myositis ossificans is the “zonal phenomenon,” which represents peripheral mineralization with central lucency (Fig. 11). Additionally, myositis ossificans is usually separated from the adjacent bone but, as the lesion matures, a stalk of attachment to the underlying bone can be seen [27].

## Pathology

**Fig. 12:**
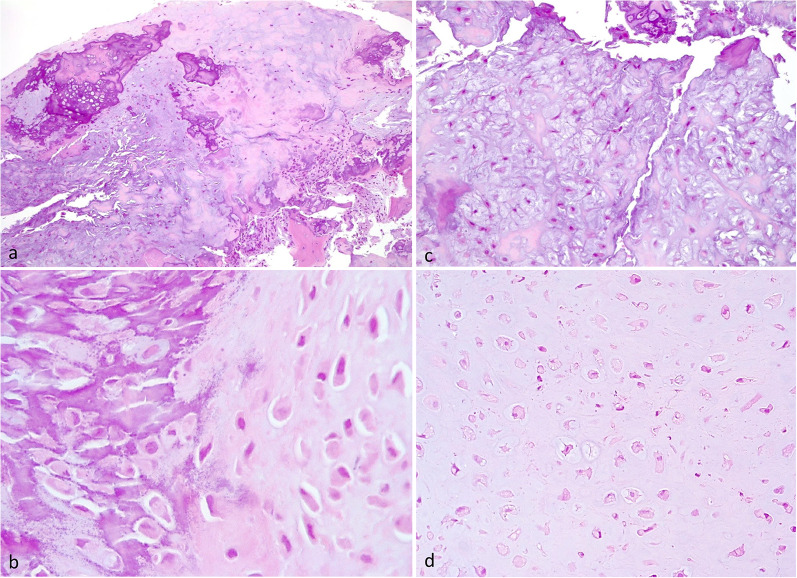
Pathological features of BPOP. Low power view of disorganized bone and cartilaginous cap (**a**). Characteristic basophilic stroma called “blue bone” (**b**). Myxoid areas with smaller spindle shaped chondrocytes (**c**). Atypical chondrocytes with hyperchromatic and enlarged nuclei (**d**)

Macroscopically, BPOP is a bony surface-based lesion with cartilaginous covering. Histologically, it is composed of a variable mixture of cartilage, bone, and fibrous tissue (Fig. 12a) [28]. The outermost part consists of hyaline cartilage and fibrocartilaginous tissue. The intermediate layer consists of cartilage-to-bone transition via enchondral ossification. The innermost part consists of trabecular bone and intertrabecular spaces containing hypervascular tissue and spindle cells. A distinguishing feature of BPOP is basophilic stroma between cartilage and bone, known as “blue bone” (Fig. 12b). The cartilage component is hypercellular and chondrocytes show atypical features, such as enlarged nuclei and binucleation (bizarre nuclei) and myxoid features (Fig. 12c, d) [2]. Differential diagnosis includes both benign—such as osteochondroma and reactive periostitis—and malignant bone surface lesions—such as chondrosarcoma and surface osteosarcoma with prominent chondroblastic component [29–32].

## Treatment and outcome

BPOP is a benign, slow-growing lesion which can be managed conservatively unless symptomatic. Treatment consists of surgical resection, which is aimed at alleviating symptoms and achieving definitive pathological diagnosis in doubtful cases. Wide resection has been proposed to reduce recurrency rates [33]. Wide resection consists of en bloc excision including the lesion with the pseudocapsule and any periosteal tissue beneath it, followed by decortication of any abnormal-appearing areas in the underlying bone [33]. However, a relatively tissue-conserving approach can be adopted in selected cases given the potential surgical morbidity associated with wide resection [34].

BPOP recurrence is relatively frequent and has been reported in up to 55% of cases, sometimes multiple times [3]. Recurrences present as partially or completely ossified masses with more irregular mineralization compared to the original lesions (Figs. 2 and 5) [7]. Recurrences are managed by re-excision [34]. BPOP has no capacity to metastasize [2].

## Conclusions

BPOP must be included in the differential diagnosis of mineralized masses arising from the periosteal aspect of bone cortex. If location and imaging features are strongly suggestive of BPOP, such as mineralized lesions arising from the phalanges with no cortical erosion or medullary involvement, a watchful waiting strategy with follow-up imaging examinations can be adopted [7]. If clinical and imaging presentation is unclear, biopsy should be performed as the next step [35, 36]. Familiarity with multi-modality imaging characteristics, pathology features and differential diagnosis is desirable to offer optimal patient care.

## Data Availability

Not applicable.

## Abbreviation

BPOP: Bizarre parosteal osteochondromatous proliferation
